# On the Use of Permselectivity to Describe the Selective Transfer of Organic Acids in Electrodialysis

**DOI:** 10.3390/membranes13060545

**Published:** 2023-05-23

**Authors:** Robin Caveriviere, Sylvain Galier, Hélène Roux-de Balmann

**Affiliations:** Laboratoire de Génie Chimique, Université de Toulouse, CNRS, INPT, UPS, 31062 Toulouse, France; robin.caveriviere@toulouse-inp.fr (R.C.); sylvain.galier@univ-tlse3.fr (S.G.)

**Keywords:** electrodialysis, separation, volatile fatty acids, ion-exchange membrane, permselectivity

## Abstract

The increasing number of investigations on the use of electrodialysis (ED) in bio-refinery requires a better understanding and tools to evaluate and describe the transfer of charged organic solutes. This study focuses, as an example, on the selective transfer of acetate, butyrate, and chloride (used as a reference), characterized by using permselectivity. It is shown that permselectivity between two anions does not depend on the total ion concentration, neither on the ion proportions, current intensity, or time nor on the presence of an additional compound. Therefore, it is demonstrated that permselectivity can be used to model the evolution of the stream composition during ED, even at high demineralization rates. Indeed, a very good agreement is found between experimental and calculated values. This study and the application of permselectivity as a tool, as developed in this paper, could be highly valuable for a wide range of applications in electrodialysis.

## 1. Introduction

The increasing demand for chemicals in various industries has led to a growing reliance on fossil fuels as a primary source of feedstock. However, the finite nature of these resources and their negative impact on the environment have necessitated the search for alternative methods and sustainable ways of production. Volatile fatty acids (VFA) are part of those basic compounds, often referred to as building blocks, widely used in industry and mostly produced from fossil resources. One of the alternative methods of production under investigation involves biological reactions. However, it requires additional steps of separation, purification, and concentration in order to recover the VFAs at the desired composition. Several separation and recovery processes have already been investigated [1]. Among them, membrane processes, such as reverse osmosis, nanofiltration [2,3], or electrodialysis [4,5,6], have the advantage of being operated without the addition of chemicals such as acids or organic solvents compared to absorption or liquid–liquid extraction processes. These separation processes are used to reach different objectives, either to purify, concentrate, or modify stream composition for further applications [7].

Conventional electrodialysis (ED) has been investigated for the bioproduction of organic acids. Associated with bioreactors, it can be used to remove inhibitors, such as acetate or lactate, and/or to extract products from the fermentation broth [4,5,8]. ED can even be directly coupled in situ with a bioreactor to recover VFAs and enhance production at a large scale [6]. A clarification step is often used to remove small particles and colloids, such as cellulose, proteins, suspended solids, or bacteria, in order to prevent the ED stack from any fouling [8,9]. Previous studies aim to investigate ED performances mostly in terms of the energy consumption or the composition of the fraction that can be recovered [9,10,11,12,13]. If the objective is to separate different compounds from one stream, permselectivity is also an interesting parameter to characterize the process performance.

However, nowadays, even though some studies are focused on the desalination of bio-refinery streams [14,15], only a few have been performed on the selectivity between VFAs with ED in order to separate them. It was shown that the transfer of VFAs decreases with their molecular weight, in the order of formate > acetate > propionate > butyrate > i-butyrate > valerate > caproate [6,16,17,18]. Permselectivity has gained increased attention with the expanding utilization of ion-exchange membranes, from desalination [19,20,21] to ion-exchange membrane bioreactors [22]. It has been shown that selectivity is mainly led by ion properties (mobility, thus size, and valence) and membrane properties [20,23,24,25,26,27,28]. Previous research about selectivity is, then, mostly focused on membrane modification to reduce water hardness for nitrate removal and selectivity toward chloride (often used as reference) or, for instance, to enhance monovalent selectivity for reverse electrodialysis [29,30,31,32,33].

In this paper, the selectivity between acetate (Ac), butyrate (Bu), and Chloride (Cl) in ED is studied. The influence of initial anion composition (concentration and proportions) and current intensity is investigated with two anion-exchange membranes and up to high demineralization rates. Additionally, this study aims to describe the evolution of solutes over demineralization with only the permselectivity, initial conditions, and electrodialysis parameters.

## 2. Materials and Methods

### 2.1. Experimental Set-Up

Experiments were realized with synthetic mixtures of sodium acetate, butyrate, and chloride at different compositions. The electrodialysis set-up is composed of a EUR 2B-10 stack (Eurodia, Pertuis, France), with a cross-section of 0.02 m^2^, 10 cell pairs *N* leading to 0.4 m^2^ of total membrane surface area, i.e., 0.2 m^2^ for each type (cation or anion-exchange membranes). Experiments were performed at constant current intensity. Diluate and concentrate compartments were initially fed with 2 L of solution each. The electrode compartment was filled with 3 L of 10 g/L of sodium sulfate. A schematic diagram of the experimental setup is depicted in a previous paper [34]. Two types of the anion-exchange membrane (Neosepta standard AMX and Neosepta selective ACS) were used with a Neosepta CMX cation-exchange membrane (Tokuyama Corp., Tokyo, Japan). Experiments have been realized below the limiting current density and with a pH between 7 and 8.

### 2.2. Analysis

Acetate and butyrate were analyzed by High-Performance Liquid Chromatography (HPLC) (Jasco LC Net II/ADC, Tokyo, Japan) equipped with an Aminex HPX-87H (BioRad, Hercules, CA, USA) column and a UV detector (UV-2077plus). The detector measured VFAs by spectroscopy with a wavelength set at 210 nm. The column temperature was set at 65 °C, and the mobile phase was a 5 mM sulfuric acid solution. The flow rate was set at 0.6 mL/min, and the injection volume varied from 2 µL to 10 µL.

Chloride was analyzed by ion chromatography (ICS 3000, Dionex, France) using an AS-11-HC column (Dionex IonPac, Sunnyvale, CA, USA) and a conductivity detector CD20. The injection volume and the temperature were set at 25 μL and 25 °C. Samples were diluted to a maximum of 200-folds by ultra-pure water (resistivity > 18 MΩ.cm) before analysis.

It has been shown in several papers that a solvent transfer appears simultaneously with the charged solute transfer [34,35]. In this study, the mass balance for water and ions was checked, knowing the variation of the volume and concentrations in the 2 compartments. The maximum deviation was less than 2% for volume and 10% for ions.

### 2.3. Experimental Procedure

It has been shown in previous works that membrane conditioning, especially membrane counter-ion, has an influence on solute and solvent transfer [36,37]. Knowing the ion-exchange capacity of the membranes used, which is lesser than 2 meq/g, and the membrane surface used, one gains a maximum of 50 meq for anion-exchange membranes AMX and ACS and a maximum of 70 meq for CMX membranes. Before each experiment, the membranes were soaked into the solution for at least 4 h with a flow rate of 180 L/h. The solution was then removed and replaced by an identical one. This procedure ensures a complete exchange in membrane counter ion, thus a fully equilibrated ion-exchange membrane (IEM). Indeed, the quantity of ions in solution is up to 10 times higher than the quantity of ions that the membrane can exchange [37].

Experiments were carried out at 24 ± 2 °C, the temperature being stabilized by an external cooling set-up. The diluate compartment was fed with different mixtures of Ac, Bu, and Cl sodium salts. Experimental conditions are listed in Table 1. The electrode compartment was fed with 3 L of a 10 g/L of sodium sulfate solution.

The concentrate compartment was initially fed with 2 L of tap water (conductivity χ = 0.3 mS/cm) to prevent high ohmic resistance that could appear with ultra-pure water at the beginning of the experiment. Electrodialysis runs were stopped either if the voltage reached U = 20 V or if the diluate conductivity reached a value lower than 5 mS/cm to prevent membrane degradation. All experiments were realized below the limiting current value. The Faradic yield, determined for each experiment, was found to remain higher than 85%. Some experiments have been conducted in triplicate, and no significant variation has been observed.

### 2.4. Chemicals

Mixtures of Ac, Bu, and Cl sodium salts have been prepared using sodium acetate (>98%, Fisher Chemical, Hampton, NH, USA), sodium butyrate (>98%, Alfa Aesar, Haverhill, MA, USA), and sodium chloride (>99.9%, VWR Chemicals, Radnor, PA, USA). Electrode compartment solution has been prepared with sodium sulfate (>99.9% VWR Chemicals, Radnor, PA, USA).

## 3. Results and Discussion

### 3.1. Theoretical Approach

Solute transfer during ED was investigated in previous studies [34,38], and the main conclusions are summarized below.

The transfer of ions in ED is the sum of two contributions [8]. The first one is migration, directly led by the current intensity. The second contribution comes from the diffusion phenomenon due to the difference in solute concentration across the membrane. It has been shown that in most ED conditions, for charged solutes, the diffusion is negligible compared to the migration [8,39]. Thus, by considering only the migration flux for the solute transfer, the quantity of ion transferred after a time *t* is given by the Equation (1), written for the diluate compartment.

(1) $$\underset{i}{\sum }z_{i}\left(n_{i}^{0}-n_{i}\right)=\eta \frac{N}{F}\;I\;t$$

In this paper, every ion valence *z* is equal to 1, and *n* and *n*^0^ are the quantities of solute (mol), respectively, at the time *t* and at *t* = 0, and *I* (A) is the applied current. The faradic yield η will be considered equal to 1. Finally, *F* (C/eq) is the Faraday constant, and *N* is the number of cell pairs, which is 10. Another parameter used to characterize the selectivity in ED is the permselectivity. Permselectivity can refer both to the selective transfer between membrane co- and counter-ions or to the selectivity between two membrane counter-ions. In this paper, the permselectivity *P_a/b_* will only refer to the selectivity between two counter-ions of the AEM, *a* and *b*, as defined by Equation (2) [20,25].

(2) $$P_{a/b}=\frac{t_{a}^{m}/t_{b}^{m}}{C_{a}/C_{b}}$$

The transport number of the ion *i* in the membrane is represented by *t_i_^m^* and the concentration in the diluate by *C_i_*. The concentration is always related to the diluate compartment. Moreover, the transport number in the membrane is defined by Equation (3) with the solute flux *J_i_* (mol/s). It represents the fraction of the current carried by the counter-ion *i* through the membrane.

(3) $$t_{i}^{m}=\frac{z_{i}J_{i}}{\sum_{j}z_{j}J_{j}}$$

The subscript j refers to all membrane counter-ions, considering that no co-ions transfer through the anion-exchange membrane. Thus, combining Equations (2) and (3), one makes the following Equation (4) for the permselectivity.

(4) $$P_{a/b}=\frac{J_{a}/J_{b}}{C_{a}/C_{b}}$$

Furthermore, solute flux is defined by Equation (5) and the concentration (mol/m^3^) by the ratio between the quantity *n_i_* and *V* (m^3^), the volume in the diluate.

(5) $$J_{i}=\frac{dn_{i}}{dt}$$

It is also supposed that permselectivity remains constant over time. This hypothesis will be validated later in the present paper. This assumption, the use of Equation (5), and the definition of the concentration ($C_{i}=\frac{n_{i}}{V}$) allows us to rewrite and integrate Equation (4) to finally achieve Equation (6).

(6) $$P_{a/b}=\frac{\ln\left(\frac{n_{a}}{n_{a}^{0}}\right)}{\ln\left(\frac{n_{b}}{n_{b}^{0}}\right)}$$

Another parameter commonly used in electrodialysis is the demineralization rate Λ. It represents the ratio between the quantity of charge transferred and the initial quantity of charge in the diluate. It is given by Equation (7) for the diluate compartment.

(7) $$\Lambda =\frac{\sum_{i}z_{i}\left(n_{i}^{0}-n_{i}\right)}{\sum_{i}z_{i}n_{i}^{0}}$$

The description of the evolution of the demineralization rate Λ, thus the quantity of solute, over time, can be determined by combining Equations (1) and (7). The resulting Equation (8) is written below.

(8) $$\Lambda =\frac{N}{F\sum_{i}z_{i}\;n_{i}^{0}}\eta \;I\;t$$

Considering two solutes *a* and *b*, the combination of Equations (6) and (7), allows to determine the variation of *n_a_* and *n_b_* versus the demineralization rate. The equations, parameters, and variables are summarized in Table 2. Python software (version 3.9.13) is used to solve the system that cannot be solved analytically.

**Figure 1:**
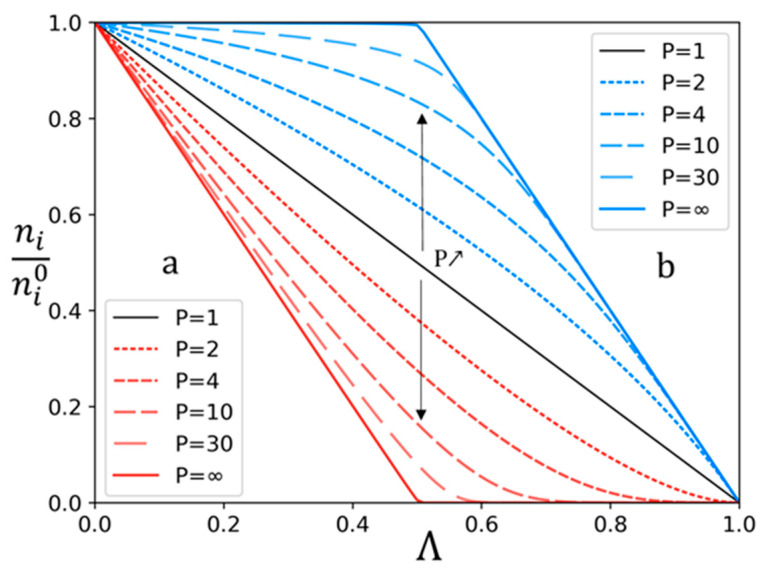
Variation of *n_i_/n_i_*^0^ of solutes *a* (red) and *b* (blue) for different permselectivities as function of the demineralization rate Λ. The initial quantity ratio of a and b is 50/50 (%mol).

To represent the evolution of the solute quantities independently of the initial quantities, $n_{a}/n_{a}^{0}$ and $n_{b}/n_{b}^{0}$ are considered instead of *n_a_* and *n_b_*. As indicated by previous equations, $n_{a}/n_{a}^{0}$ and $n_{b}/n_{b}^{0}$ depend only on permselectivity *P_a/b_*, initial solute quantities $n_{a}^{0}$, $n_{b}^{0},$ and demineralization rate Λ. An example of the variation of $n_{a}/n_{a}^{0}$ and $n_{b}/n_{b}^{0}$ versus Λ is plotted on Figure 1, for a 50/50 (%mol) mixture of two solutes *a* and *b*, and for different permselectivities.

**Figure 2:**
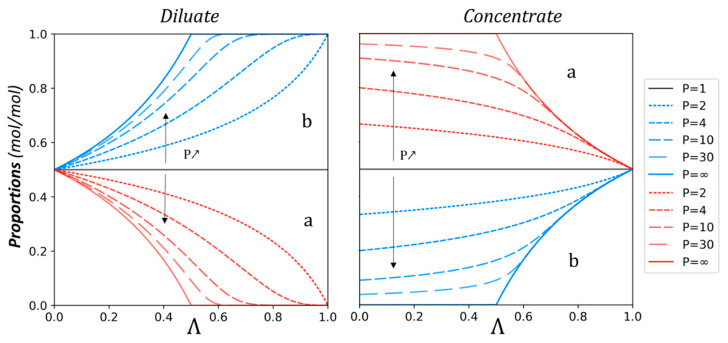
Solute proportions of *a* and *b* in the diluate and the concentrate versus the demineralization rate Λ. The initial quantity ratio of a and b is 50/50 (mol/mol).

As expected, Figure 1 shows that for a low demineralization rate, the quantity of *a* decreases faster than that of *b*. Moreover, the difference between the two anions is more important for increasing permselectivity. For a higher demineralization rate, once most of the solute *a* has been transferred, solute *b* transfer increases to ensure the transport of the applied current. This is the most visible in the case of boundary conditions, with a theoretical infinite value of permselectivity. In that case, solute *a* would be completely removed before solute *b* starts to be transferred. On the contrary, with a permselectivity of 1, i.e., no selectivity, the transfer of both solutes would be the same so that proportions would remain identical. This can be observed in Figure 2 as well, showing the evolution of the proportions of solutes *a* and *b* in the diluate and the concentrate versus the demineralization rate.

The concentrate compartment is supposed to be initially fed with water only. One can observe that the maximum proportion that can be reached in the concentrate depends on the permselectivity for a low demineralization rate. Additionally, the maximum proportion of the most transferred ion in the concentrate depends also on initial solute proportions. At the end of the process, for a demineralization rate approaching 1, all species have been transferred to the concentrate compartment, and solute proportions match the initial diluate values. Another noteworthy outcome observable in Figure 2 is that the slight permselectivity variations have a greater impact on the evolution of solute quantity and proportions on lower permselectivities. The opposite is also true for higher permselectivities.

**Figure 3:**
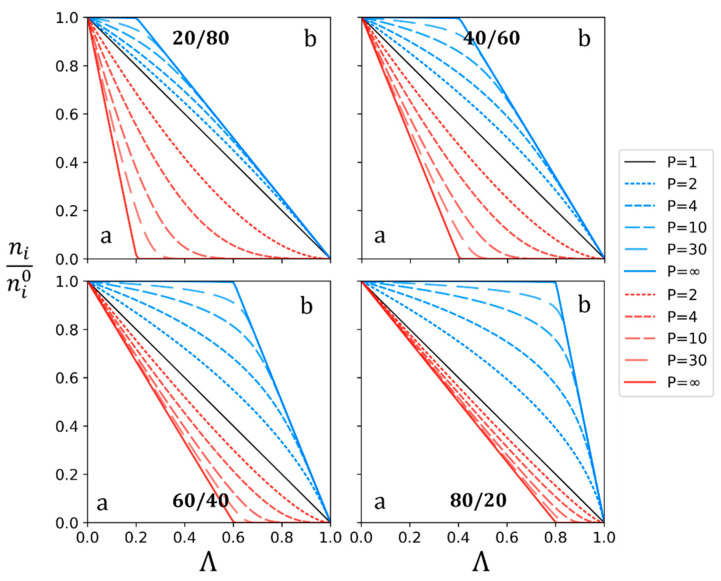
Variation of *n_i_/n_i_*^0^ of solutes *a* (red) and *b* (blue) for different permselectivities and different initial quantity ratios a/b (mol/mol), as function of the demineralization rate Λ.

Previous results were illustrated with initial identical proportions of solutes *a* and *b* in the diluate. Results obtained with different proportions are reported in Figure 3.

One can observe that the evolution of solute quantity during demineralization is heavily influenced by the initial solute quantity proportions. As the initial quantity of *a* differs from *b*, the influence of the permselectivity tends to be negligible on the solute with the highest concentration. Consequently, the influence of the permselectivity is visible on both solute quantities of *a* and *b* for a 50/50 (mol/mol) mixture, represented previously in Figure 1. Thus, this specific ratio has been chosen to design some experiments and for further representations.

Nevertheless, experimental initial *n_a_*^0^/*n_b_*^0^ ratio values can be different for every experiment realized and then can lead to different curve shapes, as shown in Figure 3. To make easier the comparison of the results obtained with various initial proportions, the evolution of the solute quantity *n_i_/n_i_*^0^ is to be set independently of *n_a_*^0^/*n_b_*^0^. To do so, values are represented as a function of the sum of individual demineralization rates Λ_i_. Additionally, to receive x-axis values between 0 and 1, the sum is divided by the number of solutes M. The resulting x-axis is named Λ^0^, and its expression is given by Equation (9).

(9) $$\Lambda^{0}=\frac{1}{M}\;\underset{i}{\sum }\frac{n_{i}^{0}-n_{i}}{n_{i}^{0}}$$

**Figure 4:**
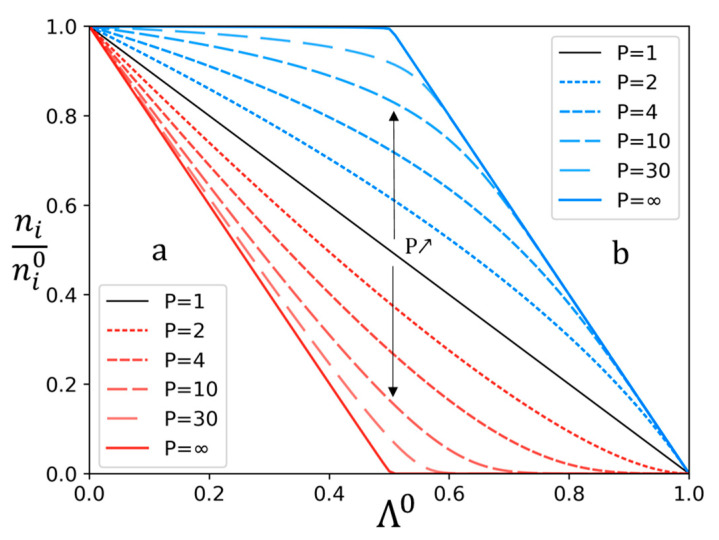
Variation of *n_i_/n_i_*^0^ versus Λ^0^ of solutes *a* (red) and *b* (blue), for different permselectivities and every *n_a_*^0^/*n_b_*^0^ value.

The representation of the variation of *n_i_/n_i_*^0^ for different ratio *n_a_*^0^/*n_b_*^0^ on Figure 3 can be then modified to fit on a single graph using Λ^0^. Hence, Figure 4 represents *n_i_/n_i_*^0^ as a function of Λ^0^.

One can observe that the representation of *n_i_/n_i_*^0^ as a function of Λ^0^ allows indeed to compare different experiments realized with a different ratio *n_a_*^0^/*n*_b_^0^. Thus, it is possible to observe the influence of other parameters on the permselectivity by using this Λ^0^ representation.

### 3.2. Experimental Study

**Figure 5:**
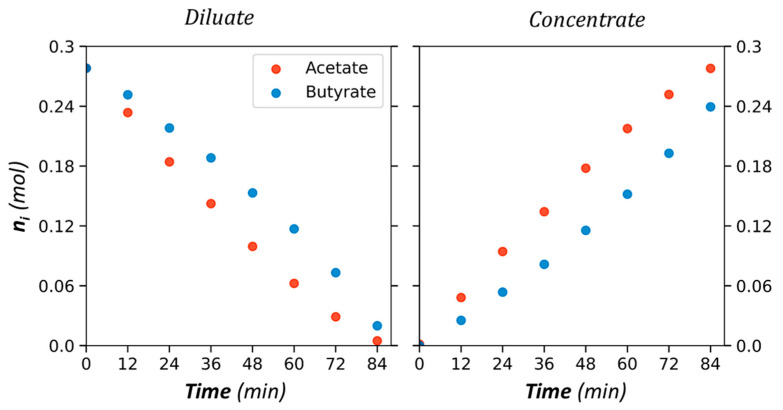
Quantity *n_i_* of acetate and butyrate versus time in the diluate and the concentrate compartment. Initial solute ratio 50/50 (mol/mol), total initial diluate anion concentration 20 g/L, AMX/CMX membranes, and current intensity I = 1A.

Experiments were carried out with different proportions and total concentrations of anions. The list of experiments is provided in Table 1. An example of a variation of quantity (mol) of Ac and Bu in both compartments is provided in Figure 5 for a 50/50 (mol/mol) composition.

**Figure 6:**
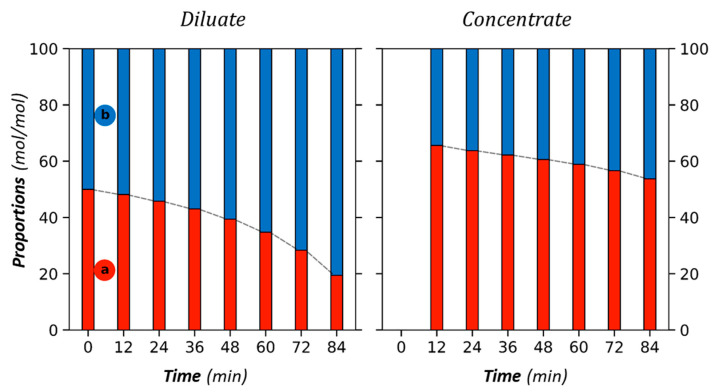
Proportion of acetate (a—red) and butyrate (b—blue) versus time in the diluate and the concentrate compartments. Initial solute ratio 50/50 (mol/mol), total initial diluate anion concentration 20 g/L, AMX/CMX membranes, and current intensity I = 1A.

These results demonstrate that acetate concentration increases faster than that of butyrate in the concentrate, despite the same initial concentrations in the diluate. Such a higher transfer of acetate compared to other VFAs in the mixture, including butyrate, was already reported in previous studies [4,6,40]. Another way to observe the selective transfer is to represent VFA proportions over time, as shown in Figure 6.

It is again found that the proportion of acetate in the concentrate is higher than in the diluate. About 65% at the beginning of the run, it decreases continuously to reach 50% (initial value in the feed) for an almost complete demineralization (95% of demineralization at the end of the operation). Simultaneously, in the diluate, the proportion of butyrate increases over time to reach 80% at the end of the operation.

**Figure 7:**
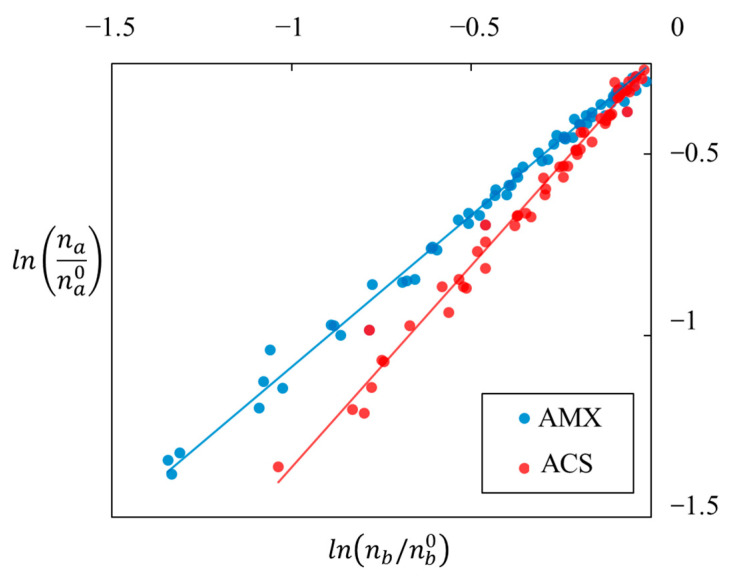
Variation of ln(*n_a_/n_a_*^0^) versus ln(*n_b_/n_b_*^0^), with a and b respectively for acetate and butyrate, and for multiple experiments with AMX and ACS anion-exchange membranes.

These results show that electrodialysis provides a selective transfer of organic acids, even with initial identical proportions and the use of standard anion-exchange membranes. To quantify this selectivity, permselectivity is determined. According to Equation (6), it is provided by the slope of the variation of $ln\left(n_{a}/n_{a}^{0}\right)$ versus $ln\left(n_{b}/n_{b}^{0}\right)$. Figure 7 shows the corresponding plots obtained with mixtures of acetate and butyrate for the two types of anion-exchange membranes used.

Data presented in Figure 6 were obtained from all experiments given in Table 1. These experiments were realized with different conditions, including current intensity, solute proportions, total anion concentration, and with or without chloride. One can observe that for a given membrane, all the points are aligned on a single straight. It means that no influence of another parameter than the membrane type can be pointed out, and a very good linear regression can be drawn for both sets of data (R^2^ > 0.99). Moreover, even for a given experiment, data shown in Figure 7 were taken at different durations, also confirming that the permselectivity does not depend on demineralization time and rate. For any set of experimental conditions, such a constant permselectivity was observed during demineralization for any couple of anions.

In a previous study, it was reported that permselectivity between sodium and magnesium ions varies with the current density. Nonetheless, a slight influence of the concentration and cation proportions was also observed [41]. These results were obtained for a couple of divalent and monovalent cations and for a different range of concentrations, demineralization rates, and current intensities. The determination of the permselectivity was also different since it was calculated considering only the initial concentrations of ions in the diluate and not their variation versus time. This approximation was made according to the range of demineralization rate investigated, which remains low enough for the transfer of ions to vary almost linearly versus time.

Permselectivity values obtained for all couples of anions are reported in Table 3 for the two anion-exchange membranes used in this work.

As expected, higher permselectivities are obtained with a more selective membrane, ACS versus AMX. The value cited as a reference and given by Astom Corporation [23] is the permselectivity between chloride and sulfate, which increases from 0.77 with AMX membrane to 2.5 with ACS. Regarding VFA value, it increases from 1.7 to 2.3 for the couple acetate/butyrate. The same statement can be made with chloride/acetate and chloride/butyrate values, which increase respectively from 5.6 to 14 and 11 to 30. Furthermore, as previously stated in the theoretical approach section, variation in permselectivity values in cases of high permselectivities results in slight fluctuations in the solute evolution. This might explain the experimental imprecisions that have been observed as permselectivities grow higher.

Moreover, from Equation (4), one draws the following Equation (10) between the permselectivity of a couple of solutes in a ternary mixture.

(10) $$P_{c/b}=P_{c/a}\times P_{a/b}$$

Using the Equation (10) for chloride/butyrate couple, for instance, permselectivity obtained are *P_Cl/Bu_* = 9.5 with standard AMX membranes and *P_Cl/Bu_* = 32 with ACS membranes. Hence, calculated values match satisfyingly experimental ones as there is an absolute deviation of respectively 1.5 and 2 for the given values compared to data shown in Table 2. The application of the previous equation on every set of values leads to the same conclusion. This supports the validity of the theoretical development but also strengthens permselectivity values obtained from experiments.

**Figure 8:**
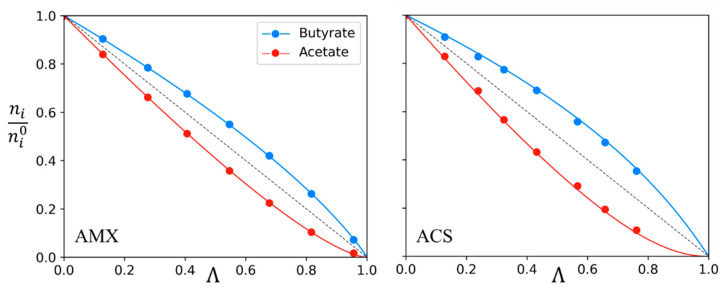
Variation of *n_i_*/*n_i_*^0^ for acetate and butyrate in the diluate versus the demineralization rate Λ. Experimental points and theoretical curves from permselectivity. Initial quantity n_Ac_^0^ = 0.28 mol and n_Bu_^0^ = 0.28 mol; *p* = 1.7, I = 1 for AMX membranes. n_Ac_^0^ = 0.25 mol and n_Bu_^0^ = 0.29 mol; *p* = 2.3, I = 1 for ACS membranes.

It has been shown previously that it is possible to determine the evolution of the quantity of solutes in a binary system, as a function of the demineralization rate, by knowing the permselectivity and the initial quantity of the solutes in the diluate. Such theoretical evolutions were plotted in Figure 1 and Figure 3 for different permselectivities and initial solutes ratios. Experimental results obtained with AMX and ACS membranes for mixtures of acetate and butyrate are compared to theoretical values and displayed as an example in Figure 8.

One can observe that there is a very good agreement between the theoretical curves determined with a permselectivity of 1.7 and 2.3 for AMX and ACS, respectively (Table 3) and the experimental points. It is again confirmed that permselectivity is constant throughout the demineralization, i.e., versus time.

**Figure 9:**
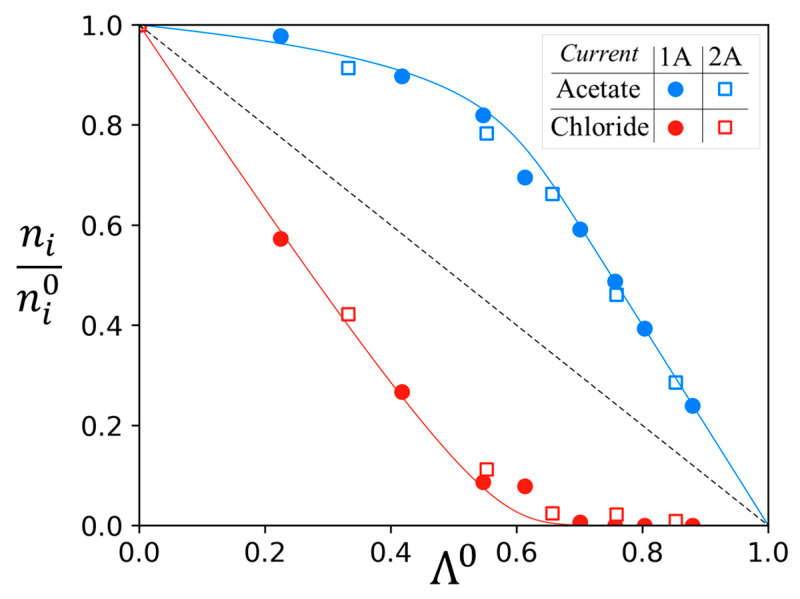
Variation of *n_i_*/*n_i_*^0^ for acetate and chloride in the diluate versus the demineralization rate Λ^0^ at 1A and 2A. Experimental data points and theoretical curves from permselectivity *p* = 14 for ACS membranes.

On the other hand, two experiments have been conducted using an initial acetate/chloride ratio of 85/15 (mol/mol), but at different current densities, respectively, 1A and 2A. Thus, the results are represented graphically in Figure 9 as a function of Λ^0^. As explained previously in the theoretical approach, all $n_{i}/n_{i}^{0}$ values for a given membrane and a given couple of solutes plotted as a function of Λ^0^ can be represented on a single curve for a specific given permselectivity value. This representation has the advantage of being independent of the initial $n_{a}^{0}/n_{b}^{0}$ ratio.

One can observe in Figure 9 that the variations of acetate and chloride for the two different experiments are very similar and fit properly with the theoretical curve, set with a permselectivity *P* = 14. Consequently, these results confirm that the current density does not influence the permselectivity for the range investigated. Experiments with higher current intensities cannot be conducted due to the resulting voltage of the system and the possibility of damaging the membranes. Thus, the range of current intensities investigated might not cover the evolution of the permselectivity approaching the limiting current or higher.

**Figure 10:**
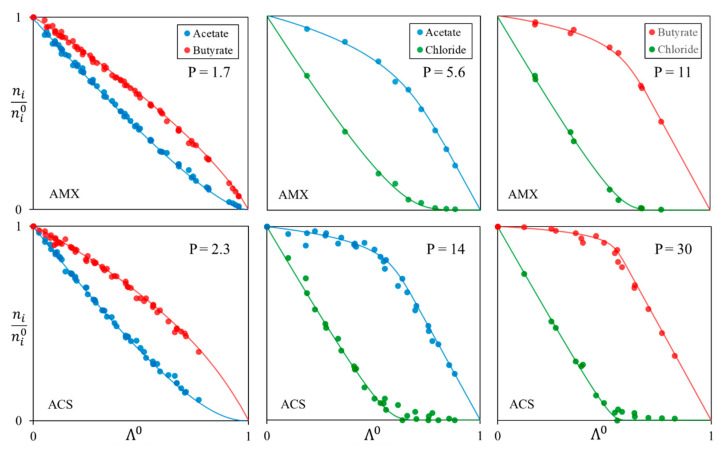
Variation of *n_i_/n_i_*^0^ versus Λ^0^ for every experiment realized, for AMX and ACS membranes, and for each solute couple. Experimental points and theoretical curves from permselectivity values.

Finally, experimental values (points) from all the experiments listed in Table 1 are reported in Figure 10, including experiments in the ternary mixture, as a function of Λ^0^. Theoretical curves are also plotted using the permselectivity values previously determined and listed in Table 3.

It is confirmed that, whatever the experimental conditions, the results obtained for a given couple of solutes and a given membrane are located on a single curve. Each calculated curve fits the experimental data satisfactorily. Thus, the mathematical expression obtained from Equations (6) and (7) describes with good agreement the behavior of two co-ions during the whole process. This also confirms that the permselectivity is independent of solute proportions, concentrations, current intensity, time (demineralization rate), and other ions in the solution.

**Figure 11:**
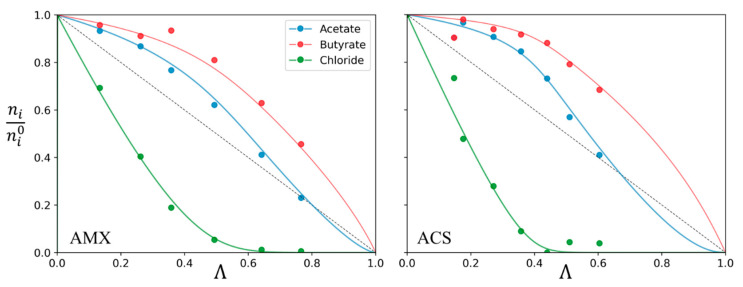
Variation of *n_i_*/*n_i_*^0^ for acetate, butyrate, and chloride as function of Λ. Experimental points and theoretical curves from permselectivity values (Table 3). Initial quantities are n_Ac_^0^ = 0.24 mol, n_Bu_^0^ = 0.21 mol, and n_Cl_^0^ = 0.20 mol for AMX membrane and n_Ac_^0^ = 0.23 mol, n_Bu_^0^ = 0.23 mol, and n_Cl_^0^ = 0.20 mol for ACS membrane.

From permselectivity values previously obtained, the system can be extended to a ternary mixture. Indeed, it has been shown previously that the permselectivity is not influenced by the other ions in the solution. In that case, the three equations needed to solve the system are the mass balance and two permselectivity equations, taking into account the three considered compounds. Data needed are still the demineralization rate and initial quantity of solutes. Figure 11 shows the evolution of $n_{i}/n_{i}^{0}$ of acetate, butyrate, and chloride in a ternary mixture as a function of the demineralization rate. Both calculated and experimental data are displayed for AMX and ACS anion-exchange membranes.

Two noteworthy outcomes can be drawn from Figure 11. First, as experimental data are in accordance with predicted values, it confirms that the permselectivity is not influenced by the presence of other compounds in the solution. Then, it also shows that the system can be solved for binary and ternary mixtures. Thus, it can be expected that as long as permselectivities are known, the system can be solved no matter the number of compounds in the solution.

## 4. Conclusions

In this work, ED was investigated for the selective recovery of VFA. Permselectivity was used to characterize the selectivity between a couple of anions, including chloride, used as the reference anion.

Experiments were carried out to study the influence of the different parameters on permselectivity. It was demonstrated that, in the range of conditions investigated, permselectivity depends only on the membrane and the solutes. Indeed, it does not depend on the concentration or proportions of anion in the feed. Moreover, permselectivity depends neither on the current intensity in the range investigated nor the demineralization rate, even for almost complete demineralization. Finally, it is not affected by the presence of a third solute. As expected, the use of more selective membranes, ACS, compared to AMX (Tokuyama Corp., Japan), increases the selectivity. The value of the permselectivity between acetate and butyrate increases from 1.7 ± 0.1 to 2.3 ± 0.1. Then, the chloride/acetate value increases from 5.6 ± 0.6 to 14 ± 3 and 11 ± 2 to 30 ± 9 for the chloride/butyrate mixture.

Permselectivity values have then been used to model the variation of the solute quantity during ED. These variations were compared with experimental ones showing a very good agreement. Since it was shown that the presence of a third anion does not change the permselectivity, this approach could be used for more complex mixtures than those investigated in this work. Furthermore, this approach can also be used in another field of study as the conclusions of this paper are not limited to volatile fatty acids. Interesting further research may be conducted on the separation of other organic solutes as well as ions of different valences, for instance.

## Figures and Tables

**Table 1:** Experimental conditions used for ED.

Anion-Exchange Membranes	Initial Anion–Molar Proportion (%)			Current Intensity (A)	Diluate Initial Total Anion Concentration (g/L)
	Acetate	Butyrate	Chloride		
AMX	93	7		1; 2; 3	20
	93	7		1	5
	93	7			40
	85	15			20
	50	50			
	33	33	33		
	50		50	2	
		50	50	1	
ACS	50	50		1	20
	50		50		
		50	50		
	85		15	1; 2	25
		85	15	1.5	
	33	33	33	1	20
	80	20			
	90	10			

**Table 2:** Equations, parameters, and variables used to determine *n_a_* and *n_b_* as functions of Λ.

Equations	Data	Variables
$\Lambda =1-\frac{n_{a}+n_{b}}{n_{a}^{0}+n_{b}^{0}}$ $P_{a/b}=\ln\left(\frac{n_{a}}{n_{a}^{0}}\right)/\ln\left(\frac{n_{b}}{n_{b}^{0}}\right)$	*n_a_*^0^ Initial quantity of *a*	*n_a_* Quantity of solute *a*
	*n_b_*^0^ Initial quantity of *b*	*n_b_* Quantity of solute *b*
	*P_a/b_* Permselectivity	Λ Demineralization rate

**Table 3:** Permselectivity values obtained from experiments for couples of acetate, butyrate, and chloride with the two anion-exchange membranes.

Permselectivity		
Membrane	AMX	ACS
Ac/Bu	1.7 ± 0.1	2.3 ± 0.1
Cl/Ac	5.6 ± 0.6	14 ± 3
Cl/Bu	11 ± 2	30 ± 9

## Data Availability

The data presented in this study are available on request from the corresponding author.

## Nomenclature

List of symbols	
C	Concentration (mol/m^3^)
F	Faraday constant (C/eq)
I	Current intensity (A)
J	Solute flux (mol.s^−1^)
M	Number of considered counter-ions
n	Quantity (mol)
N	Number of cell-pairs
P	Permselectivity
t	Time (s)
t^m^	Transport number in the membrane
V	Volume (m^3^)
z	Valence
Greek symbols	
η	Faradic yield
Λ	Demineralization rate
Λ^0^	individual demineralization rates mean
χ	Conductivity (mS/cm)
Subscripts and superscript	
Ac	Acetate
Bu	Butyrate
Cl	Chloride
i	Ion
